# Improving detection of colorectal cancer: A Bayesian approach to serial circulating tumour DNA testing following curative‐intent treatment.

**DOI:** 10.1002/ctm2.70697

**Published:** 2026-05-18

**Authors:** Susanne K. Pedersen, Geraldine Laven‐Law, Muktar Ahmed, Paul Hollington, Kathryn Cornthwaite, Benjamin L. Musher, Jean M. Winter, Erin L. Symonds, Graeme P. Young

**Affiliations:** ^1^ Flinders Health and Medical Research Institute, College of Medicine and Public Health Flinders University Adelaide Australia; ^2^ Colorectal Unit, Division of Surgery and Perioperative Medicine Flinders Medical Centre, Southern Adelaide Local Health Network Adelaide Australia; ^3^ Dan L. Duncan Comprehensive Cancer Centre Baylor College of Medicine Houston Texas USA; ^4^ Department of Gastroenterology, Division of Surgery and Perioperative Medicine Flinders Medical Centre, Southern Adelaide Local Health Network Adelaide Australia

1

Dear Editor,

Patients treated with curative intent for colorectal cancer (CRC) still have a high risk for developing metastases, resulting in poor prognosis.^1^ Detecting recurrent CRC early is therefore vital for improving outcomes.^2^ Detection of circulating tumour DNA (ctDNA) offers a valuable tool for guiding clinical decision‐making in patients with CRC.^3^ We have previously shown methylated *BCAT1/IKZF1* ctDNA (branched‐chain aminotransferase 1/Ikaros family zinc finger 1) has 66% sensitivity and 98% specificity for CRC recurrence, based on single‐point estimates.^4^, ^5^ In medical decision‐making, the likelihood of disease being present or absent evolves over time as additional diagnostic testing is conducted.^6^ Cognitive biases often contribute to diagnostic errors, such as overestimating the positive predictive value of a test when relying solely on sensitivity and specificity point estimates from cross‐sectional studies, without accounting for the prior probability of disease, which in longitudinal surveillance settings can be informed by previous test results.^7^ Bayesian updating offers a framework for interpreting and acting on test results^8^, to mitigate biases and improve diagnostic accuracy. However, there are limited data using ctDNA to trigger earlier‐than‐scheduled imaging. This study applied Bayesian updating to improve the accuracy of predicting the probability of new or recurrent CRC by incorporating prior test results with methylated ctDNA and/or carcinoembryonic antigen (CEA) test results.

Data from two studies were analysed: cross‐sectional (Figure 1A; approved by the Institutional Review Boards of participating institutes [USA])^9^ and longitudinal (Figure 1B; approved by the Southern Adelaide Clinical Human Research Ethics Committee [134.045]).^4^ Cross‐sectional: Adults who received curative treatment (stage II–III CRC) and are undergoing surveillance. Longitudinal: Patients who completed curative‐intent treatment for CRC (stages I–IV) with at least 2 years of monitoring unless a new or recurrent CRC was diagnosed sooner. Patients underwent at least one radiological assessment (gold standard for recurrence detection) with intervals between assessments determined as part of their clinical care, had at least one ctDNA test within 5 years of first CRC diagnosis (Figure S1), and their final blood test was within 12 months prior to radiological assessment. Blood samples were collected within 6 months (before or after) of radiological imaging, prior to treatment of confirmed or suspected recurrence, processed to plasma within 4 h, stored at ‐80°C for ctDNA *BCAT1*/*IKZF1* methylation‐specific qPCR^9^ and CEA testing using chemiluminescent microparticle immunoassays (Architect, Abbott Diagnostics or LIAISON, DiaSorin), where CEA ≥ 5 ng/mL was positive (Clinical Genomics Pty. Ltd. USA).

**FIGURE 1:**
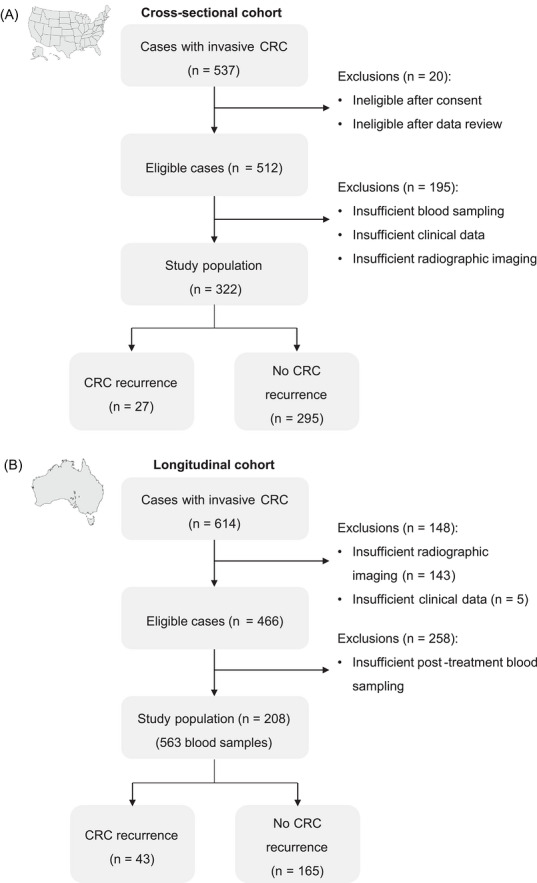
Study flow diagram. Flow diagrams for the two observational colorectal cancer patient cohorts used for (A) diagnostic accuracy point estimates in a USA cohort and (B) validation of the Bayesian approach in an Australian cohort.

Cross‐sectional outcome measures were true‐ and false‐positive rates of CEA and ctDNA for CRC recurrence. Sensitivity and specificity point estimates were calculated using frequentist statistical methods. The sensitivity and specificity point estimates of ctDNA for CRC recurrence were 0.593 (16/27) and 0.980 (289/295), respectively. CEA was detected CRC recurrence with a sensitivity and specificity of 0.481 (13/27) and 0.963 (284/295), respectively. Bayes’ formula was applied (Supporting Information Methods, Table S1) to calculate the posterior probability of ruling CRC in or out in the longitudinal cohort, based on single or serial blood tests, and to determine the number of serial blood tests (concordant or discordant; Table S1) required to reach a desired level of certainty. Posterior probability was defined as the probability of CRC being present given the observed test results. Point estimates of sensitivity and specificity used in Bayes’ formula were derived from the cross‐sectional study, along with the prior probability of CRC. The prior was varied between 0.05–0.4 to encompass a plausible surveillance risk range, based on 0.1–0.3 at the population level,^10^ to reflect different clinical scenarios. Estimates of test performance were used as fixed inputs. The predictive ability of CEA versus ctDNA was determined, relative to the assumed prior probability of CRC and the total number of blood tests. 563 opportunistically collected longitudinal blood samples were available from 208 patients (Figure 1B), with 24% (49/208) having a recurrent (*n* = 43) or new (*n* = 6) CRC (Figure S2) and 165 who remained cancer‐free (Figures S3 and S4).

The probability and observed CRC recurrence, along with CEA and ctDNA testing in the longitudinal cohort, are shown in Figure S1. Assuming a prior probability of 8.4%, consistent with the prevalence of recurrent CRC in the cross‐sectional study, individuals in the longitudinal cohort had a 73.1% probability of CRC after a single positive ctDNA, and 54.3% after a single positive CEA (Figure 2A). The probability of ruling out CRC with a single negative blood test was 96.3% for ctDNA and 95.3% for CEA (Figure 2A). Serial positive ctDNA provided significantly greater certainty of CRC being present when compared to serial positive CEA (*p* < 0.001), with a second positive ctDNA increasing the posterior probability of CRC from 73.1% to 98.8%, compared to a second positive CEA (54.3%–93.9%; Figure 2B). Serial repeat negative ctDNA testing provided more certainty of ruling out CRC compared to serial repeat negative CEA testing (Figure 2B; *p* < 0.001). Three serial concordant ctDNA tests could accurately determine diagnostic status with 99% confidence (Table 1, Figure 2B *)*. Three serial positive CEA, or four serial negative CEA, were required to reach 99% accuracy (Table 1 and Figure 2B). For discordant CEA and ctDNA results, an additional two positive tests are needed to rule in CRC with 95% confidence (Figure 2C). Conversely, if the second test is positive after the first is negative, another two negative tests are needed to rule out CRC with 95% confidence (Figure 2C). Two concordant positive CEA and ctDNA tests on the same sample calculate a 97.2% probability of CRC being present (Table 2). If a sample is CEA and ctDNA negative, the likelihood of CRC can be ruled out with 98.0% confidence (Table 2).

**FIGURE 2:**
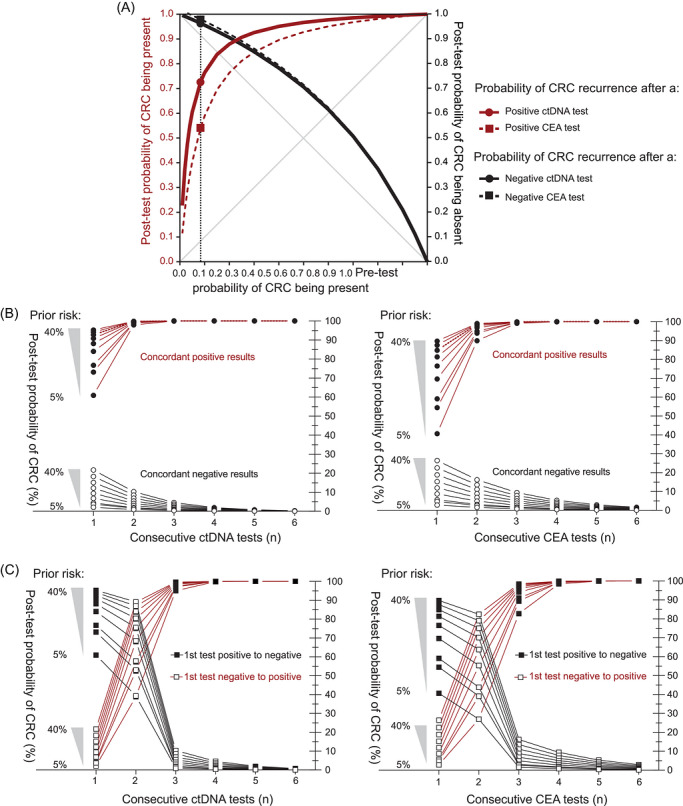
Posterior probability of colorectal cancer (CRC) being present or absent following diagnostic testing for circulating tumour DNA (ctDNA) versus carcinoembryonic antigen (CEA) using data from the longitudinal cohort. (A) Receiver operating characteristic curve demonstrating the posterior probability of CRC following a single blood biomarker test as a function of prior probability of CRC being present. The vertical dotted line corresponds to 8.4%; according to the prevalence of CRC recurrence observed in the cross‐sectional study, with accompanying probabilities for each observation denoted by shaded points. (B) Likelihood of CRC being present based on *n* consecutive ctDNA (left panel) or CEA (right panel) blood tests with concordant results. (C) Likelihood of CRC being present based on a positive (red) or negative (black) blood test followed by *n* consecutive negative (black lines) or positive (red lines) results for ctDNA (left panel) and CEA (right panel).

**TABLE 1:** Total number of diagnostic carcinoembryonic antigen (CEA) or circulating tumour DNA (ctDNA) blood tests needed (n) for a desired certainty of colorectal cancer (CRC) being present, as a function of the prior risk of CRC being present.

Probability of detecting CRC (%)	Prior likelihood of CRC being present (%)								
	5	8.4	10	15	20	25	30	35	40
	**Sequential positive CEA tests required (n)**								
**50**	2	1	1	1	1	1	1	1	1
**60**	2	2	1	1	1	1	1	1	1
**70**	2	2	2	1	1	1	1	1	1
**80**	2	2	2	2	2	1	1	1	1
**90**	2	2	2	2	2	2	2	2	1
**95**	3	3	2	2	2	2	2	2	2
**98**	3	3	3	3	3	2	2	2	2
**99**	3	3	3	3	3	3	3	2	2
	**Sequential positive ctDNA tests required (n)**								
**50**	1	1	1	1	1	1	1	1	1
**60**	1	1	1	1	1	1	1	1	1
**70**	2	1	1	1	1	1	1	1	1
**80**	2	2	2	1	1	1	1	1	1
**90**	2	2	2	2	2	1	1	1	1
**95**	2	2	2	2	2	2	2	2	1
**98**	2	2	2	2	2	2	2	2	2
**99**	3	3	2	2	2	2	2	2	2

The total number of tests is rounded to the nearest whole number.

**TABLE 2:** Computed posterior probability for ruling in or ruling out colorectal cancer (CRC) after carcinoembryonic antigen (CEA) and circulating tumour DNA (ctDNA) blood testing at a single time point.

CEA result	ctDNA result	Prior likelihood of CRC being present (%)								
		5	8.4	10	15	20	25	30	35	40
**Probability of CRC being present**										
Positive	N/A	40.6%	54.4%	59.1%	69.6%	76.5%	81.3%	84.8%	87.5%	89.7%
N/A	Positive	60.9%	73.1%	76.7%	84.0%	88.1%	90.8%	92.7%	94.1%	95.2%
Positive	Positive	95.3%	97.2%	97.7%	98.6%	99.0%	99.2%	99.4%	99.5%	99.6%
Negative	Positive	45.7%	59.4%	64.0%	73.8%	80.0%	84.2%	87.3%	89.6%	91.4%
Positive	Negative	22.1%	33.1%	37.5%	48.8%	57.4%	64.3%	69.8%	74.4%	78.3%
**Probability of CRC being absent**										
Negative	N/A	97.2%	95.3%	94.4%	91.3%	88.1%	84.8%	81.2%	77.5%	73.6%
N/A	Negative	97.9%	96.3%	95.6%	93.2%	90.6%	87.8%	84.9%	81.7%	78.3%
Negative	Negative	98.8%	98.0%	97.6%	96.2%	94.7%	93.1%	91.2%	89.2%	87.0%
Negative	Positive	54.3%	40.6%	36.0%	26.2%	20.0%	15.8%	12.7%	10.4%	8.6%
Positive	Negative	77.9%	66.9%	62.5%	51.2%	42.6%	35.7%	30.2%	25.6%	8.6%

Abbreviations: N/A; Not; applicable.

In conclusion, applying Bayesian updating to serial ctDNA and/or CEA blood tests has the potential to increase diagnostic confidence in determining whether recurrent or new CRC is present in those who have undergone curative treatment, given that three serial tests can accurately determine diagnostic status with 99% confidence. Our findings may be limited in their generalizability for serial sampling across all possible surveillance settings due to limited sample size, assumptions of test independence, opportunistic blood testing that infrequently coincided with radiographic imaging and using accuracy estimates based on a fixed time‐point. The reference tables developed here may support clinical decision‐making for radiographic follow‐up earlier than scheduled following one or more ctDNA and/or CEA blood tests.

## AUTHOR CONTRIBUTIONS

**Susanne K. Pedersen**: Conceptualisation; data curation; formal analysis; investigation; methodology; resources; visualisation; writing — original draft and writing — review & editing. **Geraldine Laven‐Law**: Data curation; investigation; visualisation; writing — original draft and writing — review & editing. **Muktar Ahmed**: Formal analysis and writing — review & editing. **Paul Hollington**: Conceptualisation; resources and writing — review & editing. **Kathryn Cornthwaite**: Conceptualisation; data curation; investigation; resources and writing — review & editing. **Benjamin L. Musher**: Investigation; resources and writing — review & editing. **Jean M. Winter**: Investigation and writing — review & editing. **Erin L. Symonds**: Conceptualisation; data curation; funding acquisition; investigation; methodology; project administration; resources; supervision and writing — review & editing. **Graeme P. Young**: Conceptualisation; funding acquisition; investigation; methodology; supervision and writing — review & editing.

## CONFLICT OF INTEREST STATEMENT

Graeme P. Young and Erin L. Symonds report research funding for the institution from Clinical Genomics Pty. Ltd. Graeme P. Young and Susanne K. Pedersen report personal fees from Clinical Genomics Pty. Ltd., NJ, USA. The other authors declare no conflict of interest.

## FUNDING INFORMATION

This study was funded in part by the National Health and Medical Research Council (APP1006242 and APP1017083), the Medical Research Future Fund (2009066) and Clinical Genomics Pty. Ltd.

## ETHICS STATEMENT

The cross‐sectional study was approved by the Institutional Review Boards of 24 participating U.S. centres. The validation cohort study was approved by the Southern Adelaide Clinical Human Research Ethics Committee (#134.045).

## PATIENT CONSENT STATEMENT

Written informed consent was obtained from all participants. The study was performed in accordance with the Declaration of Helsinki.

## Supporting information



Supporting Information

## Data Availability

The datasets analysed during this study are available from the corresponding author on reasonable request.
