# Supplementary material for: Improving detection of colorectal cancer: A Bayesian approach to serial circulating tumour DNA testing following curative‐intent treatment
Source: Clin Transl Med. 2026 May 18;16(5):e70697. doi: 10.1002/ctm2.70697 (PMC13181328; doi:10.1002/ctm2.70697)
Supplement: Supplementary file 1 — Supporting Information [file CTM2-16-e70697-s001.docx]

**Supporting Methods:**

The posterior probability for recurrent colorectal cancer (CRC) being present or absent after a single or multiple positive blood test was based on the following Bayesian equation;

Equation S1: $P\left( D+ | T+ \right)=\frac{Sn \times\varphi}{Sn \times\varphi+\left( 1-Sp \right) \times(1- \varphi)}$

where $P\left( D+ \right| T+)$ denotes the probability of a positive blood test result where CRC is present, Sn denotes test sensitivity, Sp denotes test specificity, and $\varphi$ denotes the prior probability of CRC being present; spanning from 0 (no risk of CRC) to 1 (certain CRC), and encompassing one or more risk factors.

The posterior probability estimates of CRC being present following $n$ serial positive blood tests was adapted from Balayla *et al.*:

Equation S2: $P\left( D+ | T+n \right)=\frac{{Sn}^{n} \times\varphi}{{Sn}^{n} \times\varphi+ \left( 1-Sp \right)^{n} \times\left( 1- \varphi\right)}$

The number of consecutive positive blood test results required for a desired level of certainty ($p$) of CRC being present was calculated by rearranging Equation S2:

Equation S3: $n_{Positive tests}=\frac{\ln\left[ \frac{p \varphi-p)}{\varphi p-\varphi} \right]}{\ln\left[ \frac{Sn}{1-Sp} \right]}$

The posterior probability of CRC being absent following a negative test ($P\left( D- | T- \right)$) was calculated based on the following formula:

Equation S4: $P\left( D- | T- \right)=\frac{Sp \times(1 - \varphi)}{Sp \times\left( 1 - \varphi\right) + (1-Sn) \times\varphi}$

The posterior probability estimates of CRC being absent following *n* serial negative blood tests was calculated as follows:

Equation S5: $P\left( D- | T-n \right)=\frac{{Sp}^{n} \times(1 - \varphi)}{{Sp}^{n} \times\left( 1- \varphi\right)+ \left( 1-Sn \right)^{n} \times\varphi}$

The number of consecutive negative blood test results required for a desired level of certainty of CRC being absent was calculated by rearranging Equation S5:

Equation S6: $n_{Negative tests}=\frac{\ln\left[ \frac{- \varphi p}{(1- \varphi)( p-1)} \right]}{\ln\left[ \frac{Sp}{1-Sn} \right]}$

Equations S2 and S5 were used to calculate the posterior probability following any two blood tests (Table S1).

**Table S1.** Bayesian equations for calculating the posterior probability of colorectal cancer (CRC) being present or absent or after two blood test results.

| **Equation name** | **Disease status** | **Test result** | **Formula** |
| --- | --- | --- | --- |
| Equation S7 | CRC present ($D+$) | Two serial negative results ($T--$) | $\frac{{(1-Sn)}^{2} \times\varphi}{{(1-Sn)}^{2} \times\varphi+ \mathrm{Sp}^{2} \times(1-\varphi)}$ |
| Equation S8 | CRC absent ($D-$) | Two serial positive results ($T++$) | $\frac{{(1-Sp)}^{2} \times(1-\varphi)}{{(1-Sp)}^{2} \times(1-\varphi) + \mathrm{Sn}^{2} \times\varphi}$ |
| Equation S9 | CRC present ($D+$) | Two discordant results ($T+-$) | $\frac{Sn\times(1-Sn) \times\varphi}{Sn\times(1-Sn) \times\varphi+ Sp\times(1-Sp) \times(1-\varphi)}$ |
| Equation S10 | CRC absent ($D-$) | Two discordant results ($T+-$) | $\frac{Sp\times(1-Sp) \times(1-\varphi)}{Sp\times(1-Sp) \times(1-\varphi) + Sn\times(1-Sn) \times\varphi}$ |
| Sn; test sensitivity, Sp; test specificity, $\varphi;$ prior probability of CRC being present (encompassing one or more risk factors). | | | |

**
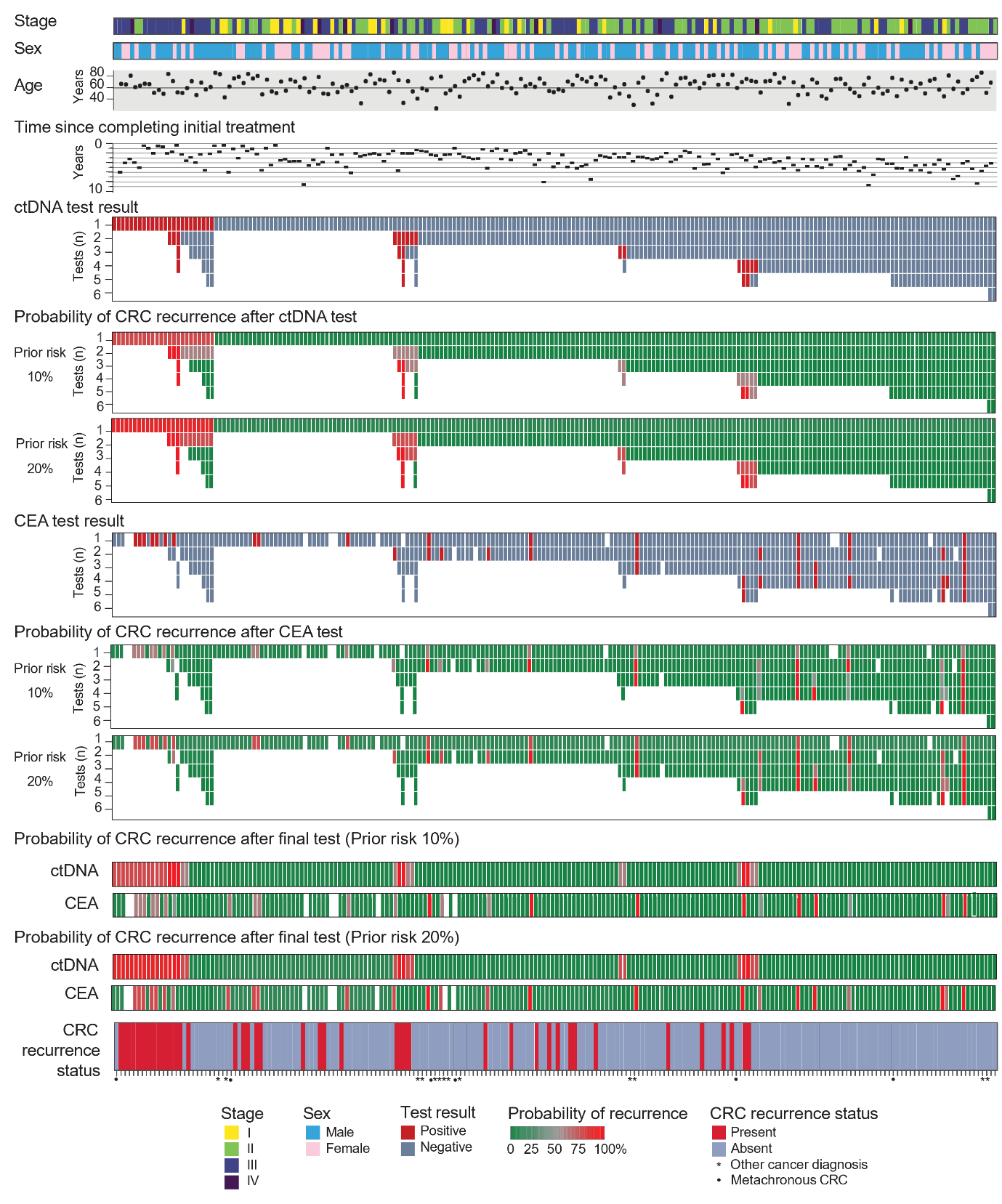
**

**Supplementary Figure S1. Likelihood of colorectal cancer (CRC) being present with sequential circulating tumor DNA (ctDNA) or carcinoembryonic antigen (CEA) testing and the observed CRC recurrence in the longitudinal cohort.** Heatmap shows patient demographics and the duration of follow-up since completing initial treatment for n = 208 patients, alongside their corresponding ctDNA and CEA result (where available) for *n* consecutive blood tests. Posterior probability estimates based on both a 10% and 20% prior probability of CRC are provided for each patient and each new result as a sliding window based on their previous test, alongside with final CRC status.

**
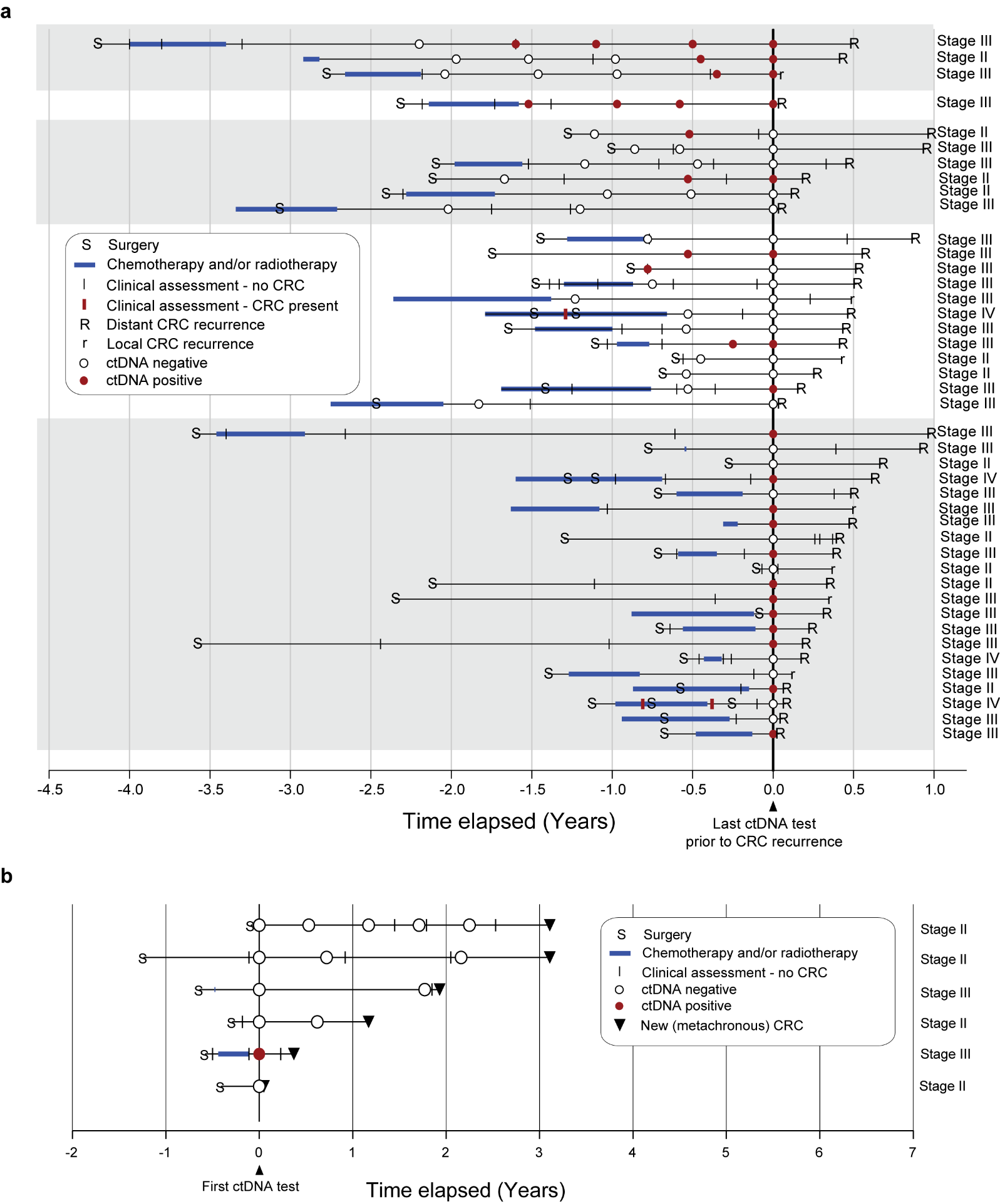
Supplementary Figure S2.** Swimmer plot showing the clinical timelines for all patients within the longitudinal cohort with a new or recurrent CRC over the study period. **(a)** n = 43 patients with colorectal cancer (CRC) recurrence, **(b)** n = 6 patients with metachronous CRC. ctDNA; circulating tumor DNA.

**
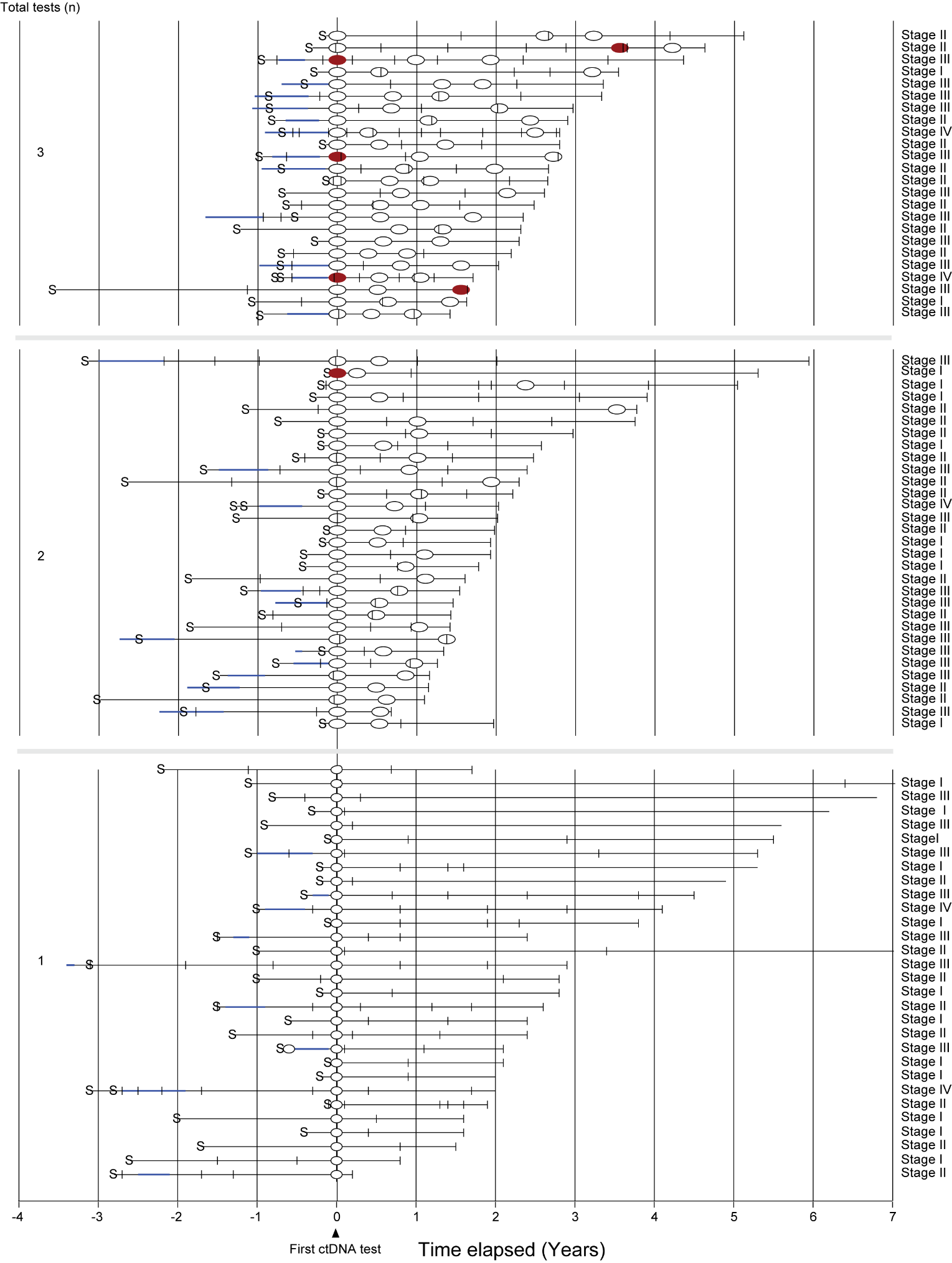
Supplementary Figure S3.** Swimmer plot showing the clinical timelines for n = 85 patients who remained free from colorectal cancer (CRC) who have undergone up to three circulating tumor DNA (ctDNA) tests within the longitudinal validation cohort.

**
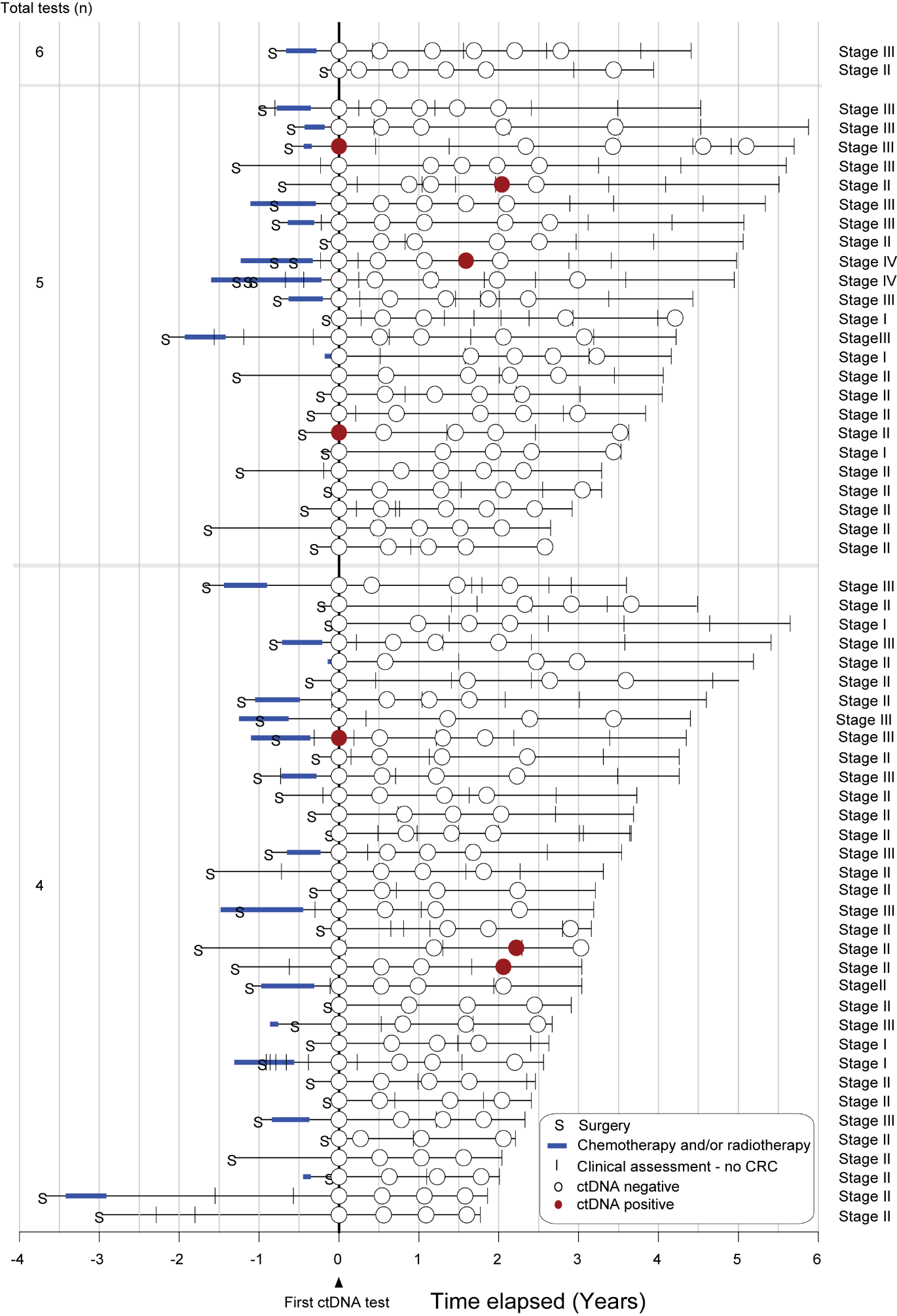
Supplementary Figure S4.** Swimmer plot showing the clinical timelines for n = 60 patients who remained free from colorectal cancer (CRC) who have undergone four to six circulating tumor DNA (ctDNA) tests within the longitudinal validation cohort.
